# Autologous Bone Marrow Mononuclear Cells Intrathecal Transplantation in Chronic Stroke

**DOI:** 10.1155/2014/234095

**Published:** 2014-07-08

**Authors:** Alok Sharma, Hemangi Sane, Nandini Gokulchandran, Dipti Khopkar, Amruta Paranjape, Jyothi Sundaram, Sushant Gandhi, Prerna Badhe

**Affiliations:** ^1^Department of Medical Services and Clinical Research, NeuroGen Brain and Spine Institute, Stemasia Hospital and Research Centre, Plot No. 19, Sector 40, Near Seawoods Railway Station, Nerul, Navi Mumbai, Maharashtra 400706, India; ^2^Department of Research and Development, NeuroGen Brain and Spine Institute, Stemasia Hospital and Research Centre, Plot No. 19, Sector 40, Near Seawoods Railway Station, Nerul, Navi Mumbai, Maharashtra 400706, India; ^3^Department of Neurorehabilitation, NeuroGen Brain and Spine Institute, Stemasia Hospital and Research Centre, Plot No. 19, Sector 40, Near Seawoods Railway Station, Nerul, Navi Mumbai, Maharashtra 400706, India

## Abstract

Cell therapy is being widely explored in the management of stroke and has demonstrated great potential. It has been shown to assist in the remodeling of the central nervous system by inducing neurorestorative effect through the process of angiogenesis, neurogenesis, and reduction of glial scar formation. In this study, the effect of intrathecal administration of autologous bone marrow mononuclear cells (BMMNCs) is analyzed on the recovery process of patients with chronic stroke. 24 patients diagnosed with chronic stroke were administered cell therapy, followed by multidisciplinary neurorehabilitation. They were assessed on functional independence measure (FIM) objectively, along with assessment of standing and walking balance, ambulation, and hand functions. Out of 24 patients, 12 improved in ambulation, 10 in hand functions, 6 in standing balance, and 9 in walking balance. Further factor analysis was done. Patients of the younger groups showed higher percentage of improvement in all the areas. Patients who underwent cell therapy within 2 years after the stroke showed better changes. Ischemic type of stroke had better recovery than the hemorrhagic stroke. This study demonstrates the potential of autologous BMMNCs intrathecal transplantation in improving the prognosis of functional recovery in chronic stage of stroke. Further clinical trials are recommended. This trial is registered with NCT02065778.

## 1. Introduction

Recovery after stroke is quite heterogeneous, as it is determined by the site and extent of lesion. The process of recovery occurs through a combination of spontaneous and learning-dependent processes. These include restitution (restoring the functional status of the injured neural tissue), substitution (reorganization of the spared pathways in order to relearn the lost functions), and compensation (reducing the disparity between the impaired function of the patient and the environmental demands on the patient) [1]. The process by which the above events occur includes angiogenesis, neurogenesis, and synaptic plasticity [2].

The overall outcome of these patients improves with rapid diagnosis, early preventive treatment, early recognition of complications, and mobilization [3]. After a central nervous system (CNS) insult like stroke, the quiescent stem cells present in the bone marrow show increased mobilization and migration from their resident bone marrow into the blood circulation as a result of cytokine production from the CNS. Thus the recruitment of bone marrow cells in the injured area of the brain is amplified. This process is the driving factor for the natural recovery process [4]. However, as the chronic stage approaches, the number of mobilized cells reduces, thus decreasing the rate of recovery. Most of the rehabilitative approaches are based on the concept of neuroplasticity and cortical reorganization of the available neurons after injury, thus assisting in the natural pattern of functional recovery [5, 6].

Cell therapy has the potential to induce all of the above neurorestorative processes. Stem cells are advantageous for therapy due to their multipotency; they can be multiplied in vitro and grafted into the developing and mature CNS [7–9]. Adult bone marrow is a rich source of stem cells. The stem cells derived from bone marrow have a great potential as therapeutic agents in the management of stroke. They are easily obtainable and can be expanded easily ex vivo for autologous transplantation [2]. Also they can be differentiated into neurons on exposure to various inducing regimens and are capable of secretion of growth factors, which are critical for neuronal survival [10–13]. There are mounting evidences which support the capacity of bone-marrow derived cells to mobilize from the bone marrow into the peripheral blood, eventually homing into the injured brain. This action is epitomized in bone marrow stem cells mobilization following an ischemic brain injury [4]. There are various clinical trials conducted including animal and human models, which aimed at assessing the effects of cell therapy in stroke. The changes observed after the cell transplantation in the animal studies included increased angiogenesis, increased modulation of neurotrophic growth factors, and reduction in the infarct volumes [14–20]. Human studies showed increased neural plasticity, improved functional recovery, and restoration of neurological deficits. There was enhanced angiogenesis as a result of increased levels of endogenous VEGF and other neurotrophic factors [21, 22].

There is a need to analyze the effect of cell therapy in patients with chronic stroke. In this study, we have studied effect of autologous BMMNCs in 24 patients with chronic stroke, who had reached a plateau phase.

## 2. Materials and Methods

### 2.1. Study Design

**Figure 1:**
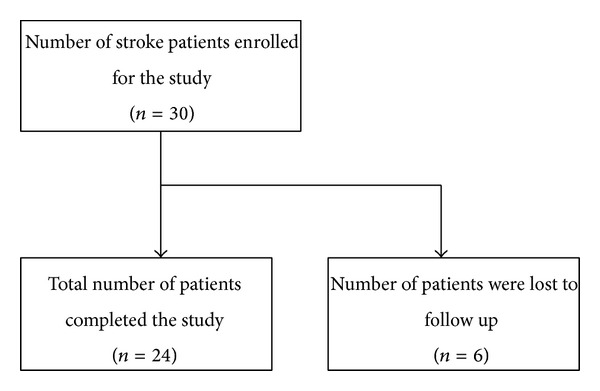
Procedure for patient selection.

The objective of the study was to analyze if the transplantation of autologous BMMNCs along with neurorehabilitation helps in improving the functional level of the patients, along with balance, hand functions, and ambulation. A nonrandomized sample of 30 patients with chronic stroke was included in the study. Out of these, 6 patients were lost to follow up and thus they were not included in the analysis; see Figure 1.

### 2.2. Patient Selection

Patients were selected based on the World Medical Association Declaration of Helsinki: ethical principles for medical research involving human subjects [23]. The protocol of the study was approved by the Institutional Committee for Stem Cell Research and Therapy (IC-SCRT) in accordance with the Indian Council of Medical Research (ICMR) guidelines. The inclusion criteria were male and female patients, age group of 18 and above, diagnosed as stroke by clinical presentation and MRI findings. Exclusion criteria were presence of respiratory distress, presence of acute infections such as HIV/HBV/HCV, malignancies, and other acute medical conditions such as respiratory infection, fever, hemoglobin less than 8, bleeding tendency, bone marrow disorder, left ventricular ejection fraction <30%, and pregnancy or breastfeeding. The intervention was performed after gaining written informed consent from all the patients/relatives in case of affection of higher mental functions.

### 2.3. Preintervention Assessment

Before the cell transplantation, all the patients underwent a comprehensive neurological evaluation. They were assessed for standing and walking balance, hand functions, and ambulation. Functional assessment was performed on functional independence measure (FIM), whereas higher mental function was tested on Mini Mental State Examination (MMSE). All the patients underwent routine biochemical, serological, and hematological tests for medical fitness. If the patients were on anticoagulants, they were stopped 48 hours prior to the procedure and restarted 48 hours after the procedure.

### 2.4. Isolation of BMMNCs

All the patients were administered granulocyte-colony stimulating factor injection 48 hours and 24 hours prior to the procedure, to mobilize the cells and to enhance their numbers. Bone marrow aspiration was carried out under local anesthesia with or without sedation, depending on the individual case. Around 120 mL of bone marrow was aspirated from the right anterior superior iliac spine using bone marrow aspiration needle and collected in heparinized tubes. Mononuclear cells (MNCs) were obtained by density gradient separation. The isolated MNCs were checked for viability manually and confirmed on cell count machine by propidium iodide. The average viability count was 96%. The MNCs were checked for CD34+ by fluorescence activated cell sorting (FACS) using CD34 PE antibody.

### 2.5. Administration of BMMNCs

The separated MNCs (body weight (kg) × 10^6^) were administered immediately after separation. Using a spinal needle, the thecal sac was punctured in the L4-L5 lumbar space. The cells were then injected through the spinal needle. The patients were on intravenous methylprednisolone 1 gm in 500 mL Ringer Lactate during the procedure of cell transplantation, to avoid local reaction to the cells.

### 2.6. Neurorehabilitation

After cell transplantation, every patient underwent an individualized neurorehabilitation program designed according to each patient requirements consisting of physiotherapy, occupational therapy, and psychological interventions.

### 2.7. Monitoring and Follow-Up

Patients were monitored regularly for any immediate adverse effects in the hospital for 4 days after the cell therapy. The patients were advised for regular follow-up at 3 months and 6 months and yearly thereafter. During each follow-up, the patients underwent complete neurological assessment and were monitored for any long-term adverse effects. The patients were followed up for minimum of 6 months to maximum of 4.5 years. 

*Outcome Measures.* All the patients were assessed on FIM. They were also assessed for changes in ambulation, hand functions, and standing and walking balance.

## 3. Results

**Figure 2:**
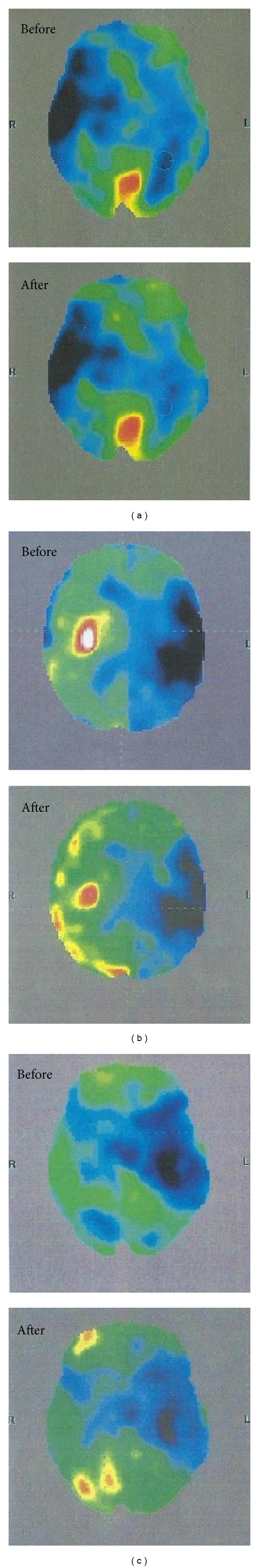
PET scans of 3 patients before and after cell therapy. Increased FDG uptake is seen in all 3 patients in the postintervention PET-CT scans. The blue areas represent hypometabolic areas, green areas represent normal metabolic activity, and black areas represent absent metabolic activity (primary area of stroke). All 3 images show reduction in the blue areas and increase in the green area, showing improved metabolic activity. (a) Increased FDG uptake is seen in left parietal and temporal lobes indicating increased metabolic activity. (b) Increased FDG uptake is seen in the left frontal, temporal, parietal, and occipital lobes, along with left hippocampus, parahippocampus, and left amygdale. (c) Increased FDG uptake is seen in left frontal, parietal lobes. There is also increased FDG uptake in the right parietal lobe.

24 patients diagnosed with chronic stroke were included in this study based on the set inclusion and exclusion criteria. The demographic characteristics of all the patients are described in detail in Table 1. The stroke territory lesion of these patients included frontal: 2 patients, temporal: 1, frontoparietal: 1, temporoparietal: 4, temporooccipital: 1, parietal occipital: 1, frontotemporoparietal: 4, frontal temporal parietal occipital: 2, thalamic: 1, gangliocapsular: 4, BG, thalamus, pons, and corona radiata: 1, frontal temporal parietal occipital, BG, and mesial temporal: 1, and pons, BG, and cerebellum: 1. All the patients underwent autologous BMMNCs intrathecally. Following cell therapy, patients underwent individualized neurorehabilitation. None of the patients had any major adverse events. Out of 24 patients, 3 patients agreed to undergo positron emission tomography-computerised tomography (PET-CT) imaging before and after cell therapy. The PET scans are shown in Figure 2.

### 3.1. Statistical Analysis

Descriptive statistics were used to analyze the demographic characteristics of the patients; see Table 1. Patients were grouped according to age, type of stroke, time since stroke, and side of brain involvement. As the number of patients in each subgroup was low, percentage analysis was performed to find the percentage of improvement symptomatically and functionally in each group. Statistical analysis was done using Wilcoxon signed rank test to compare the pre- and post-FIM values. This test was performed using the SPSS 20.0 version; see Table 3.

Results showed that, overall out of 24 patients, 12 improved in ambulation, 10 in hand functions, 6 in standing balance, 9 in walking balance, and 10 patients in functional status; see Table 2. Patients with age less than 60 years showed a higher percentage of improvement in the areas of ambulation, hand functions, and sitting and standing balance, as compared to the patients with age more than 60 years. They also showed improvement in the FIM scores. Time since the stoke episode also seemed to have an effect on the recovery of patients. The percentage of improvement was higher in patients, whose episode of stroke was less than 2 years old, as compared to those patients whose stroke was older than 2 years. Patients with ischemic type of stroke had better outcomes in all the mentioned areas, as compared to those with hemorrhagic stroke. Also, patients with right brain involvement showed higher percentage of improvement in area of ambulation, standing balance, and walking balance, as compared to the left brain; see Table 2.

**Figure 3:**
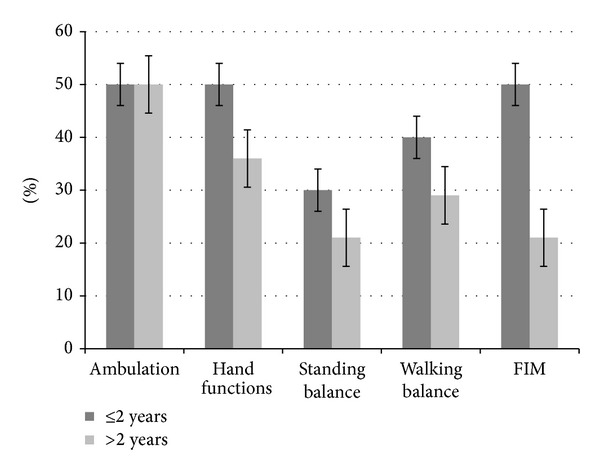
Graph representing comparison of symptomatic improvement and functional improvement in patients of ≤2 and >2 years of onset of stroke.

**Figure 4:**
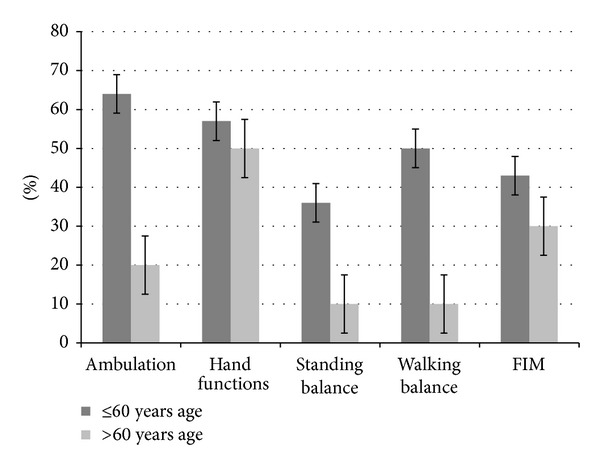
Graph representing comparison of symptomatic improvement and functional improvement in patients with age ≤60 and >60 years.

**Figure 5:**
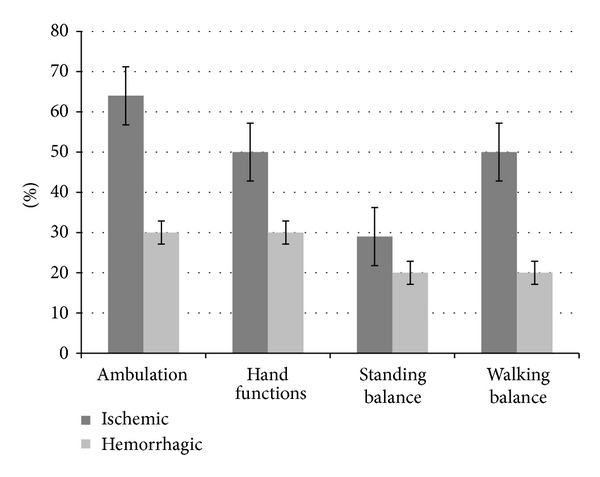
Graph representing comparison of symptomatic improvement in patients with ischemic and hemorrhagic stroke.

**Figure 6:**
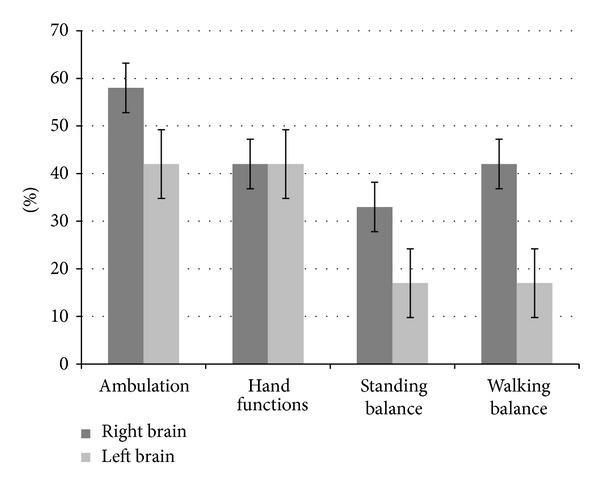
Graph representing comparison of symptomatic improvement in patients with the involvement of left and right brain.

With respect to higher mental functions, out of total, 9 patients were affected. Two patients showed improvement in higher mental functions after cell therapy and neurorehabilitation. There was a statistically significant difference (*P* < 0.05) seen in FIM scores before and after the cell therapy. The results are depicted in Figures 3, 4, 5, and 6.

## 4. Discussion

Initially, it was considered that CNS does not possess the capacity to regenerate and neurorestorative processes are not possible after an insult to the CNS. With time, the understanding of brain had greatly advanced, with properties like neuroplasticity proving that brain is capable of regeneration and restoration. Thus, it is critical to search more novel and therapeutic approaches which can be administered in the chronic phase of stroke and which would amplify the intrinsic properties of the brain for neuroplasticity and the neurological recovery [24].

### 4.1. Rationale for Use of Cell Therapy in Stroke

Neurogenesis, angiogenesis, and synaptic plasticity, which lead to the process of neurorestoration, contribute to the functional recovery after stroke. It is seen that adult rodent brain generates neuronal progenitor cells in the subventricular zone (SVZ) and in the dentate gyrus of the hippocampus throughout the life of the animal. Endogenous precursors are a source for neuronal replacement after brain injury, which is suggested by the persistence of neurogenesis in the adult mammalian brain. After stroke, the neuroblast population expands greatly in the SVZ. These cells are then recruited to the penumbral area, which is the area bordering the infarct, where they can differentiate into neurons, thereby replacing the lost neurons [25, 26]. The neuroblasts may also act synergistically with the microvasculature leading to stimulation of angiogenesis and synaptic activity in the local environment, thus promoting neurological recovery [24]. After brain injury, synaptic plasticity is related to behavioral change and functional recovery [27]. The potential morphological strategies which enable the brain to reorganize the neuronal circuits consist of increased dendritic arborization [28]. After a stroke, the synaptic activity increases at the penumbral area. This is evidenced by increase in the expression of synaptic proteins such as synaptophysin and growth associated protein. The production of these proteins is enhanced with successful neurorestorative treatments [29]. In response to injury, such as stroke, the quiescent stem cells of bone marrow get mobilized resulting in increased migration from their resident bone marrow into the blood circulation. A key chemokine implicated in the quiescence of these cells within the bone marrow is stromal derived factor-1 (SDF-1), which acts via its major receptor CXCR-4 (C-X-C chemokine receptor type 4). This pathway is essential for the stem cell migration and seeding. When SDF is activated, these stem cells are activated and are released in the circulation. The CNS then contributes to the mobilization of these cells by cytokine production, which is amplified in stroke. Thus the recruitment of these cells in the brain is increased [4].

### 4.2. Mechanism of Action of BMMNCs in Stroke

The mechanism of BMMNCs mimics the natural process of recovery after stroke as discussed above. The regenerative potential of bone marrow stem cells, a type of stem cells, has been validated in various studies, including brain ischemia [30]. There are also few studies done which showed improvement in the long-term functional outcomes in patients with stroke as a result of increase in brain plasticity, after delayed treatment with stem cells [31–33]. Bone marrow stem cells are multipotent and can differentiate into different tissue types, including astrocytes, neurons, and endothelial cells in the brain [14, 34]. These cells secrete various growth factors like VEGF, bFGF, and BDNF which promote functional outcome after stroke. These growth factors support and amplify angiogenesis, neurogenesis, and synaptic plasticity at the penumbral region [17, 35, 36]. Along with the above neuroreparative processes, the MNCs also decrease the glial scar formation and promote glial-axonal remodeling. Thus the MNCs act in a pleiotropic way to stimulate brain remodeling [32].

### 4.3. Source, Selection, and Route of Administration

Bone marrow mononuclear cells were the chosen type, as they are the most easily accessible and most studied source of stem cells. Initially the bone marrow was thought to contain only hematopoietic type of stem cells. However, increasing amount of evidence has shed light on this concept. The bone marrow cells comprise hematopoietic stem cells, tissue-specific progenitor cells, stromal cells, and specialized blood cells in different stages of development. These cells differ in their potential to differentiate and form cells giving rise to tissues which are different from the main stem cell [37]. Since they are autologous, use of these cells does not hold the risk of any graft versus host diseases or tumors. These cells were introduced intrathecally into the patients system. Intrathecal route was used for administration of cells, as it is a minimally invasive technique. It has also been found that administering intrathecally through the CSF may be a more targeted way of transmission than intravenous [38].

Cell therapy has been shown to improve functional status in many of the neurological disorders, including cerebral palsy, mental retardation, spinal cord injury, and stroke as well [39–46]. There is a randomized controlled trial of long-term effects of autologous mesenchymal stem cell transplantation in patients with ischemic stroke. A randomized controlled trial was performed in the year 2010 by Lee and colleagues, to assess the long-term effects of transplantation of autologous mesenchymal stem cells in patients with middle cerebral artery infarct. A total of 52 patients were allocated randomly to mesenchymal stem cells (MSC) treated group and into a control group and were followed up for 5 years. When compared with the control group, the follow-up modified Rankin Scale (mRS) score was decreased, whereas the number of patients with a mRS of 0–3 increased in the MSC group. Clinical improvement in the MSC group was associated with serum levels of stromal cell-derived factor-1 and the degree of involvement of the subventricular region of the lateral ventricle. The authors thus concluded that intravenous autologous MSCs transplantation was safe for stroke patients during long-term follow-up [22].

Here, overall patients showed improvements in the areas of ambulation, hand functions, and standing and walking balance and function. These improvements can be contributed to the physiological processes occurring at the microcellular level in the brain as a result of cell therapy. The neurorestorative effects exerted by the BMMNCs like angiogenesis, neovascularisation, production of growth factors, and paracrine effects lead to increased synaptic plasticity at the penumbral area in the brain. These processes help in the formation of neuronal maps, which are strengthened with neurorehabilitation. In the present study, all the patients had undergone standard methods of treatment available and still demonstrated the residual deficits before undergoing cellular therapy. It was observed that there are a few factors, which affect the recovery of patients with stroke. Factors like age, time since stroke, type of stroke, and side of involvement of the brain were analyzed, to see if they had any influence on the recovery after stroke. It was observed that patients with age less than 60 years of age showed higher percentage of improvement with respect to ambulation, hand functions, standing and walking balance, and FIM scores, as compared to the patients older than 60 years. The causes for lesser recovery in aged population are multifactorial. It has been exhibited in the past that the number of stem cells derived from bone marrow declines with age. There is also an age related decline in the “fitness” of these cells, which might affect their usefulness in remodeling of the CNS [47]. Aging process is associated with progressive losses in function across multiple systems including sensation, cognition, memory, motor control, and affection. In older adults, there are 4 core factors which interact with each other, therefore creating a downward spiral of degraded brain functions. These 4 factors include reduced schedules of brain activity, noisy processing, weakened neuromodulatory control, and negative learning [48]. Age related changes in brain plasticity have also been proven in the past. The neurotransmitter interactions in functionally connected systems may change as a function of age. Glutamate has a role in the maintenance of cellular function, as well as cell death which reduces with age. Several glutamatergic transporters and receptors play a critical role in synaptic and dendritic plasticity [49]. Along with affected balance and increased instability as a result of ageing, older patients are less likely to perform vigorous exercises, thus directly affecting the quality of rehabilitation [50]. Previous studies have shown that younger patients had better survival and early and long-term outcomes [51].

Time since stroke was another factor analyzed in this study. The group of patients, whose time after stroke was less than 2 years, showed better improvement in all the areas, as compared to the ones who had the stroke episode more than 2 years. There are studies which state that earlier intervention in patients with stroke shows better outcomes with respect to functional gains on FIM measurement [52]. As the time after the stroke increases, there is an alteration in the muscles with respect to structure and function. Muscular atrophy is seen, with shift to fast myosin heavy chain in the hemiparetic leg muscles, leading to alterations in the gait patterns. As the time progresses, contractures and deformities set in, which leads to altered biomechanical alignment, directly affecting the rehabilitation. At the microcellular level, after injury to the neurons, inflammatory processes begin, with time leading to scarring at the neuronal level. Thus the plasticity of the brain reduces, making it difficult to learn new patterns by the process of neuronal mapping. At the physiological level it has been postulated that stem cells along with G-CSF induced mobilization of CD34 cells may have contributed to the angiogenesis, which provided a supportive microenvironment for neuronal regeneration, and that both the angiogenesis and the neurogenesis by stem cells along with G-CSF treatment play roles in functional restoration during chronic stroke [53]. Along with neurogenesis and angiogenesis, MSCs significantly decrease the glial scar formation, promoting glial-axonal remodeling [32].

Stroke is classified into 2 main categories, depending on the pathophysiology-ischemic and hemorrhagic stroke. It has been seen in the literature that hemorrhagic strokes are more severe than the ischemic strokes and overall have poor outcomes, due to larger hemorrhage volume. In hemorrhagic stroke, rupture of the vessel leads to extravasation of the blood into the neighboring brain tissue, thus affecting the surrounding areas of the brain and the dampening of their functions, along with the primary hemorrhagic area. In contrast, ischemic stroke seems to have better outcomes, as a result of collateral blood circulation of the neighboring areas of the infarct, thus maintaining the tissue viability and preventing expansion of the injury [54]. We postulate that, in the hemorrhagic stroke, more numbers of stem cells may be needed for remodeling of the widespread injury, as compared to the ischemic stroke. The findings of our study correlate well to the above events. Patients with ischemic stroke showed better outcomes in the form of higher percentage of improvements with respect to ambulation, hand functions, standing and walking balance, and FIM scores.

Side of involvement of the brain may have an effect on the outcome of patients with stroke. In a study, it has been shown that the total functional scores of patients with right brain affected were slightly higher at the time of admission and discharge than for the ones with left brain affected in all the age groups. The possible reasons for the lesser improvement in patients with left brain affection was due to statistically significantly lower subscores for communication and social cognition, most likely affected by aphasia [55].

A study done by Hicks et al. in 2007 demonstrated the role of physical activity and enriched environment on the recovery of stroke models when administered with stem cell therapy. Enriched housing and voluntary running exercise enhanced migration of transplanted stem cells toward the region of injury after stroke and there was a trend toward increased survival of stem cells. Enrichment also increased the number of endogenous progenitor cells in the subventricular zone of transplanted animals. Functional recovery measured in the cylinder test was facilitated only when the stem cell transplants were combined with enrichment and running exercise 7 days after the transplant. These results suggest that the ability of transplanted stem cells in promoting recovery can be augmented by environmental factors such as rehabilitation [56].

In this study, all the patients were given a choice of undergoing 18-fluorodeoxyglucose (FDG) PET CT scans. Out of 24 patients, 3 patients agreed to undergo PET CT scan before and after cell therapy in a gap of 1 year. PET CT scan is a noninvasive functional imaging tool, which examines the correlation of changes in the metabolic activity of the brain with the activity of the nervous tissues. The 18-FDG dye used for the PET CT scan is an analogue of glucose which provides functional information of the cells based on the glucose uptake. There are glucose transporter proteins, which transport FDG to the cells. This is then metabolized and converted into FDG-6 phosphate, which cannot be metabolized further and gets trapped into the cell, as the cell membrane is impermeable to this molecule. This trapping of the glucose molecule is considered to be directly proportional to the rate of glycolysis in the tissue. Reduced FDG uptake indicates reduced metabolic activity [57, 58]. The standardized uptake value (SUV) is used as a relative measure of FDG uptake [59]. These values are compared to a normal controlled SUV and a standard deviation (SD) value is calculated which indicates the areas of the brain functioning beyond the normal limits. Hence, in stroke the ischemic areas which are hypofunctioning are seen as areas of hypometabolism. Increased FDG uptake in hypofunctioning areas indicates improvement. We hypothesize that the paracrine effects, neoangiogenesis, and secretion of various growth factors lead to improved oxygenation and functioning of damaged neurons, leading to increased glucose uptake, thus increasing the metabolic activity of the ischemic areas (see Figure 2).

## 5. Limitations

The limitations of this study are that it is a nonrandomized, uncontrolled single-center study with a small sample size and heterogeneous groups. Also, effective dose and timing of cell therapy need to be studied. Effect of repetition of cell therapy dose on the recovery of patients with chronic stroke needs to be analyzed. Magnetic labeling of the injected cells would be helpful to analyze the homing of these cells in the injured brain. PET-CT was available only for 3 patients out of 24. We would have got a better insight of the changes at the microcellular level if the investigations were available for the complete sample. Though this study has shown improvement in the chronic stage, it is difficult to attribute it to either cell therapy or rehabilitation individually.

## 6. Conclusion

This is a preliminary study, which supports the use of combination of cell therapy and neurorehabilitation. It also indicates that intrathecal autologous BMMNCs transplantation is safe, feasible, minimally invasive, and a viable option. It may be beneficial in accelerating the functional recovery in patients with chronic stroke and enhance the results of rehabilitation. Thus, by augmenting the process of recovery, after all the available treatment options are exhausted, cellular therapy fulfills the current unmet medical need in chronic stroke. Earlier intervention may influence the amount of recovery in patients with chronic stroke. Factors like time since stroke and type of stroke may also influence the process of recovery. Though the intrathecal administration of autologous BMMNCs augments the recovery process in chronic stage of stroke, which has come to a plateau, it does not help in complete recovery. Thus our quest of finding different type of cells, route administration, and dose is ongoing.

## Figures and Tables

**Table 1:** Demographic characteristics.

Age	Minimum	27
	Maximum	79
	Mean	57

Gender	Males	15 (62.5%)
	Females	9 (37.5%)

H/O diabetes	Present	8 (33.3%)
	Absent	16 (66.7%)

H/O hypertension	Present	15 (62.5%)
	Absent	9 (37.5%)

Type of stroke	Ischemic	14 (58%)
	Hemorrhagic	10 (42%)

Time since stroke (months)	Minimum	4
	Maximum	144
	Mean	40.54
	Std dev.	31.29

Follow-up period (months)	Minimum	6
	Maximum	54 (4 and half years)
	Mean	30 (2 and half years)

**Table 2:** Percentage analysis of effect of each factor on the recovery of 24 patients with stroke (*n* = 24) (% of improvement) (values in brackets indicate number of patients improved).

	Total patients improved (*n* = 24)	Time since stroke		Age		Type of stroke		Side of involvement of brain	
		<2 yrs (*n* = 10)	>2 yrs (*n* = 14)	<60 yrs (*n* = 14)	>60 yrs (*n* = 10)	Ischemic (*n* = 14)	Hemorrhagic (*n* = 10)	Right (*n* = 12)	Left (*n* = 12)
Ambulation	(12) 50	(5) 50	(7) 50	(9) 64	(2) 20	(9) 64	(3) 30	(7) 58	(5) 42
Hand functions	(10) 42	(5) 50	(5) 36	(8) 57	(5) 50	(7) 50	(3) 30	(5) 42	(5) 42
Standing balance	(6) 25	(3) 30	(3) 21	(5) 36	(1) 10	(4) 29	(2) 20	(4) 33	(2) 17
Walking balance	(9) 38	(4) 40	(4) 29	(7) 50	(1) 10	(7) 50	(2) 20	(5) 42	(2) 17
FIM	(9) 38	(5) 50	(3) 21	(6) 43	(3) 30	(5) 36	(4) 40	(3) 25	(6) 50

**Table 3:** Comparative analysis of FIM in patients before and after cell therapy (*N* = 24).

	Mean pre-FIM	Mean post-FIM	Significance (*P* < 0.05)
FIM score	73.67	78.17	0.007
